# Remote Recognition of Moving Behaviors for Captive Harbor Seals Using a Smart-Patch System via Bluetooth Communication

**DOI:** 10.3390/mi12030267

**Published:** 2021-03-04

**Authors:** Seungyeob Kim, Jinheon Jeong, Seung Gi Seo, Sehyeok Im, Won Young Lee, Sung Hun Jin

**Affiliations:** 1 Department of Electronic Engineering, Incheon National University, Incheon 22012, Korea; scape111@inu.ac.kr (S.K.); jjh0806@inu.ac.kr (J.J.); 202023053@inu.ac.kr (S.G.S.); 2 Division of Polar Life Sciences, Korea Polar Research Institute, Incheon 21990, Korea; alex0127@kopri.re.kr

**Keywords:** biologging system, harbor seals, remote data capturing, Bluetooth communication

## Abstract

Animal telemetry has been recognized as a core platform for exploring animal species due to future opportunities in terms of its contribution toward marine fisheries and living resources. Herein, biologging systems with pressure sensors are successfully implemented via open-source hardware platforms, followed by immediate application to captive harbor seals (HS). Remotely captured output voltage signals in real-time mode via Bluetooth communication were reproducibly and reliably recorded on the basis of hours using a smartphone built with data capturing software with graphic user interface (GUI). Output voltages, corresponding to typical behaviors on the captive HS, such as stopping (A), rolling (B), flapping (C), and sliding (D), are clearly obtained, and their analytical interpretation on captured electrical signals are fully validated via a comparison study with consecutively captured images for each motion of the HS. Thus, the biologging system with low cost and light weight, which is fully compatible with a conventional smartphone, is expected to potentially contribute toward future anthology of seal animals.

## 1. Introduction

In the recent past, animal telemetry has been dramatically developed [1,2,3], and its importance in terms of key achievements is highly recognized due to its contribution toward marine fisheries and living resources. Moreover, technological advances which render remote sensing possible for animal species such as sharks, tunas, salmon, sturgeon, marine mammals, reptiles, and seabirds have been significantly expedited [4,5,6,7,8,9,10,11,12,13,14,15], leading to preferable accumulation of high-quality biological information and oceanographical observation during commute through ocean habitats. From this perspective, a biologging system is a core factor in the enhancement of an in-depth understanding of biological/physiological information, corresponding to body temperature, heart rate, blood or tissue oxygen saturation, tailbeat, sound, etc., and oceanographic variables such as pressure, light, temperature, salinity, and position, and biology [16].

Thus, with respect to biologging, multiple types of sensors have been applied to various animal taxa from insects to birds and mammals, and the number of papers on biologging subjects reached over 600 publications in 2014 [17]. Recent techniques enable miniaturizing the multiple sensors to a mass of 1–2 g such that even small-sized flying birds can be tracked to collect information of light intensity, atmospheric pressure and temperature, and acceleration [18]. Furthermore, for behavioral studies, acceleration provides locomotory information by interpreting three-dimensional movement at a resolution over 10 Hz [19]. This provides specific body posture or energy expenditure, which was limited in previous observational field studies. Thus, it especially gives opportunities to researchers to record swimming behaviors even when underwater by categorizing them into detailed movements [20].

Just as wild animals have been widely studied to understand their natural behavior and ecology, captive animals are also good model systems to apply initial biologging methods [21,22,23]. Biologging techniques require the retrieval of sensors, unless they have a function allowing information transfer via satellites or other stationary receivers. However, there are possible losses of data sensors if not recaptured in the wild within a limited time. Therefore, researchers have tried deploying newly developed sensor platforms, leading to a minimization of possible data loss when monitoring captive animals. Furthermore, captive animals are important for education, genetic conservation, and rehabilitation [24]. In this sense, in zoos and aquariums, the development of animal-borne sensors and their application toward captive animals are highly required to enhance the quality of daily health and wellbeing in real time [25]. Furthermore, such technologies may contribute to animal welfare in captivity, as well as behavioral monitoring.

In this study, we aimed to measure electric signals on captive harbor seals (HS) (*Phoca vitulina*). Harbor seals are widely distributed along the Arctic coastlines of the Northern Hemisphere, and, among the five subspecies, the Ungava seal (*Phoca vitulina mellonae*) is listed as endangered on the International Union for Conservation of Nature (IUCN) red list [26]. For remote recognition of behaviors for the seal individual, we estimated the body movement and tested a new platform using a remote sensor system on a marine mammal in captivity. Therefore, the present development platform for the captive sensor system can be potentially applied to captive animals, leading to securing platforms which can be potentially beneficial for wild animals.

## 2. System Architecture

**Figure 1:**
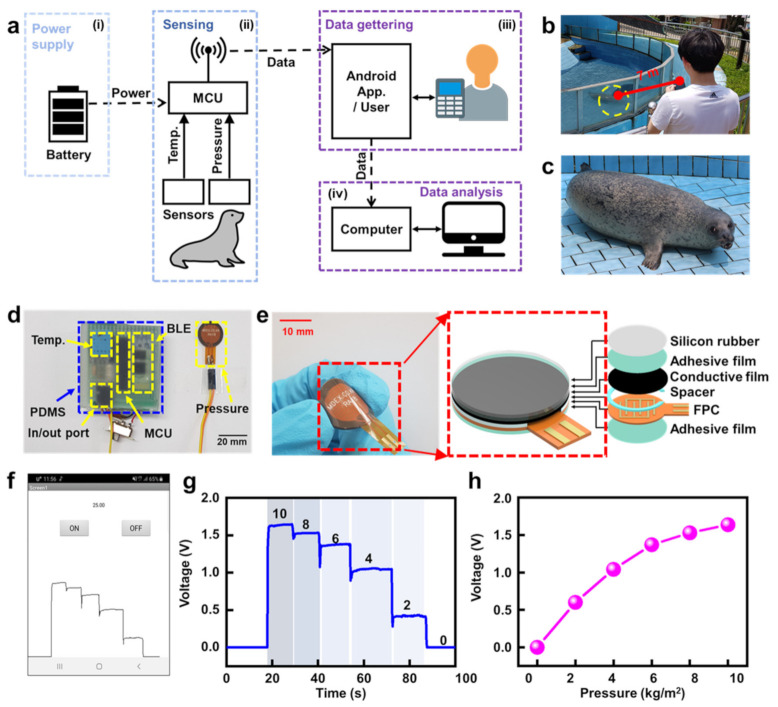
(**a**) Block diagrams for remote recognition systems: (i) power supply allowing the monitoring of moving behaviors on captive harbor seals (HS) via wireless data gathering through Bluetooth communication to a smartphone (user), (ii) microcontroller unit connected to sensors, (iii) example of data gathering using an Android application, and (iv) computer systems for data analysis. (**b**) Real-time image capturing the gathering of sensing data from captive harbor seals (distance ≈ 7 m). (**c**) Posture image of HS. (**d**) Optical images for implemented remote recognition systems, encapsulated with polydimethylsiloxane (PDMS) for waterproofing. (**e**) Optical image for flexible pressure sensors and exploded views of their assembly. (**f**) Representative image showing smartphone screen during the real-time monitoring of sensing data. (**g**) Graph of voltage vs. time during real-time monitoring. (**h**) Output voltage of pressure sensor, corresponding to various pressures ranging from 0 to 10 kg/m^2^.

Figure 1a illustrates block diagrams of the remote recognition system, comprising microcontroller units with various sensors, data gathering parts using an Android application, data analysis using a computer, and a power supply to monitor moving behaviors on the captive HS. Figure 1a(i) shows the power supply unit using a 3.7 V LiPo battery, which renders all systems remotely operational without having an externally wired power connection. Figure 1a(ii) describes the sensing units, which enable simultaneously detecting two vital signals of body temperature and pressure on the captive HS in real time via temperature and pressure sensors attached to the HS. As shown in Figure 1a(ii), HS indicates one of the targeted objects which need to be monitored. In this work, a captive HS was selected as one of the main vehicles to demonstrate the capability of remote recognition of vital signals with miniature biologging systems built using conventional Bluetooth communication platforms. In particular, microcontroller units (MCUs) play a key role in converting the captured analog signals via sensors in the HS to digital signals. Moreover, MCUs manage operational principles and their logic in various sensors attached to the HS. Figure 1a(iii) illustrates the data gathering process, which involves transmitting all converted digital signals from MCUs to observers using conventional smartphones with the Android operational system (OS), leading to the data being saved on smartphones. During the transmission of digital signals, all communication protocols are based on Bluetooth communication, which enables encompassing the Bluetooth communication distance (class 2) of 10 m between receivers and transmitters [27,28,29,30], as well as the communication interval of 160 ms for each access. In addition, all captured data are visually displayed and can be saved according to end-user purposes on a smartphone using a graphic user interface (GUI) implemented with the Android (OS). Thereafter, all data saved on the smartphone can be easily handled for transmission to personal data storage or/and cloud computers for real-time data analysis and meaningful data mining [31]. For the validation of a reasonable conclusion regarding the remotely captured data and their physical meaning, real-time video information using a smartphone was recorded for motion capture of the HS during remote monitoring of the behaviors of the captive HS [10,11,32,33,34,35].

Figure 1b shows the image captured by the observer recording (or saving) videos (or transmitted data) displaying the behaviors of the HS. The observer was located at a distance of 7 m from the monitoring subject (i.e., the captive HS), and adequate data transmission and data saving using a smartphone were confirmed without jamming. Figure 1c shows an image captured displaying the posture of the captive HS during remote monitoring of its behaviors. Figure 1d shows an optical image of the implemented remote recognition systems, which were fully protected with waterproof sealing using polydimethylsiloxane (PDMS) [36,37,38] encapsulants for monitoring the captive HS. Figure 1e shows an optical image of a pressure sensing unit including an exploded view of the detailed assembly, composed of a pressure sensor, silicon rubber, adhesive film, conductive film, spacer, flexible printed circuit (FPC), and adhesive film. The pressure sensor, attached to the flippers of the captive HS, was intentionally designed for quantitative data capture describing the direction, amplitude, and frequency of flippers movement according to various motions such as stopping, rolling, flapping, and sliding. Figure 1f shows a captured image of voltage signals in real-time mode displayed on the screen of a smartphone during monitoring. Figure 1g,h show graphs of captured voltage versus time and output voltage values as a function of pressure with a step of 2 kg/m^2^, ranging from 0 kg/m^2^ to 10 kg/m^2^.

### 2.1. Hardware

#### 2.1.1. Background on Sensors for Motion Capture 

In the recent past, the most widely used method for motion tracking or motion capture on human beings was achieved using either physical sensors (e.g., gyroscopes) attached to the subject’s body or total package systems based on infrared rays and their detection devices, leading to the analysis of accurate motion [39,40,41,42]. Furthermore, previously reported motion tracking systems were very effective in the detection of human motion in real time because they were based on an accurate passive marker or active marker system commonly used in motion capture sensors, yielding direct data interpretation for complex physical (or anatomical) movements and their interactions without any additional computation. Thus, it is magnificently faster than conventional manual work, leading to a significant improvement of work efficiency in understanding motion capture and its physical meaning [42,43,44,45,46]. However, these schemes have fundamental limitations when the same platform is applied to sea animals in an open-water environment, which are uniquely violent and difficult to anesthetize, presenting large hurdles for the attachment (or detachment) of sensors to sea animals. In parallel, even if the attachment of sensors to specific animals is successful, most animals in the wilderness will not retain the sensors on specific body parts. With respect to additional requirements, multiple cameras, which enable detecting signals from passive or active sensors attached to the targeted sea animals, describing each motion of the subjects, need to be prepared. Therefore, complicated (or bulky) sensors and optical systems including cameras, which inevitably require a stable posture without agitation, are tremendously challenging in terms of their establishment in the wilderness due to the requirement for frequent reestablishment corresponding to each physical moment of the targeted subjects, at least on the scale of meters.

However, in the present work, the proposed system, which enables detecting movement, direction, and amplitude of the targeted animals according to sensor signals from pressure sensors, is highly beneficial for understanding muscle movement and its output in wild animals. This is attributed to main platforms which can detect a rate of change while subjecting animals to relatively less stress [47,48,49,50,51,52,53,54]. In this work, to overcome the limitations of conventional methods (e.g., passive or active markers), a strain measurement system was implemented using RA18 devices (Marveldex inc., Korea), which are commercially available, to detect muscle changes. The basic characteristics and structure of the RA18 pressure sensor are shown in Figure 1e,f. Considering that the acceleration-based approaches are being used to categorize animal behaviors into a few simplified categorization, this method is expected to produce a similar level of behavioral estimation.

#### 2.1.2. Specifications of Microcontroller Units (MCUs) 

For the implementation of biologging systems with versatility, which enables adding (or eliminating) various sensors according to each design, board design platforms for MCUs are convenient. In this work, all analogous outputs from pressure sensors were designed for direct connection to the input nodes of an ATmega328P (~16 MHz). However, there are many tricky aspects when implementing the function of a boot loader with only one unit of ATmega328P. Thus, the development of microcontroller boards (e.g., Arduino Uno R3 board, which is well established as an open-source hardware platform) was utilized as an immediate and easy solution in terms of functionality for boat loading in MCUs. Then, after inserting the chip into the Arduino Uno R3 board, the software was immediately uploaded via a USB serial connection to the host PC or laptop provided by Arduino [55]. After removing the chip from the board after programming, the programmed logic and operational principles, according to their associated operation sequences, were saved to be recalled at any time in a nonvolatile mode, followed by adequate execution of the required action, by simply connecting the power supply to the entire system.

**Figure 2:**
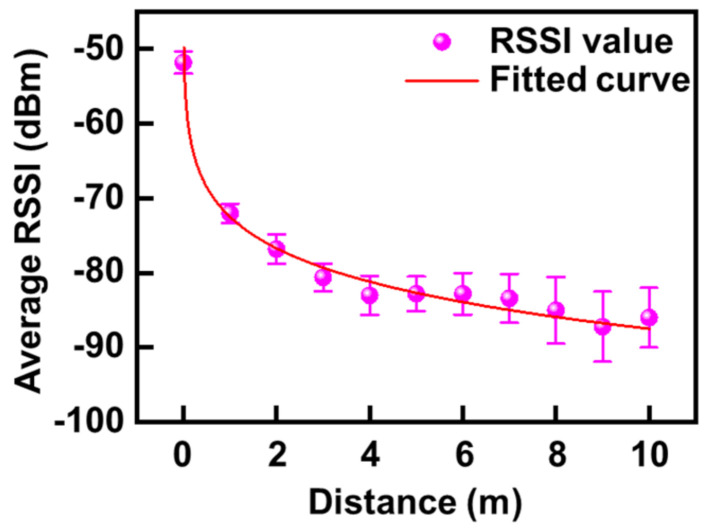
Curve fitting for received signal strength indicator (RSSI) values at distances of 0–10 m in an open environment.

In addition, the HC-06 module was selected as the wireless communication module between the smartphone and ATmeage328P because it requires a relatively low operating voltage, similar to the voltage range (3.3–4.2 V) required for ATmeaga328P [56]. Moreover, resources for the HC-06 module (Guangzhou HC Information Technology Co., Guangzhou, China) were enough to provide motion detection for each sensor. Figure 2 shows the values of received signal strength indicator (RSSI) between the HC-06 module and the smartphone as evidence of proper operation.

For reliable data acquisition, five consecutive measurements under the same experimental conditions were performed and all data were gathered, followed by statistical analysis, as shown in Figure 2. The average RSSI monotonically decayed as physical distance increased, with a physical constant of distance (~6.4 m) extracted at the 1/e value of −84.5 dB·m when considering an initial value at 0 m.

#### 2.1.3. Estimation of Power Consumption

**Figure 3:**
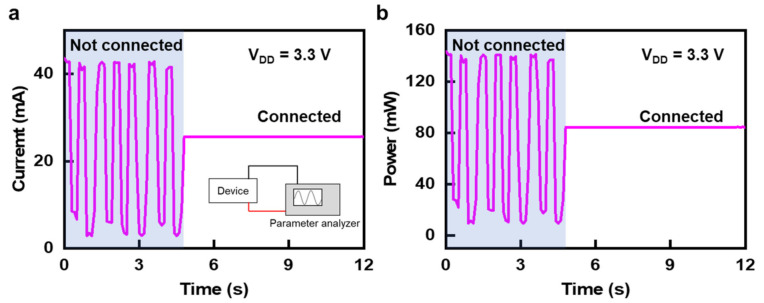
Power consumption of assembled devices with measurement scheme: (**a**) current versus on time and (**b**) power versus time with an applied bias (V_DD_) of 3.3V.

Figure 3 and Table 1 show the required current level and power consumption of assembled devices in the system for its adequate operation. In particular, the pressure sensor and temperature sensor have very high resistance, with the consumed current converging to zero. The total current consumption is 25 mA at an input voltage of ~3.7 V, and the operating time can be expressed as follows [57]:

(1) $$t=\frac{C}{I^{k}}$$

where *t* is the operating time, *I* is the discharge current, *C* is the battery capacity, and *k* is Peukert’s coefficient. For a realistic estimation of battery lifetime, the value of *k*, according to battery chemistry and the manufacturing process, is adopted in a typical range from 1.1 to 1.3. With substitution of the battery specifications (3.7 V, 350 mA) used in this system, the total operational time guaranteeing proper operation of the biologging system with a fully charged battery capacity was estimated as 7.74 ± 2.4 h. However, one time duration, right after fully charging the battery, can be potentially adjusted by the improvement of battery capacity or increase of number of battery if required for a specific application.

### 2.2. Software Platforms for Biologging System

For the establishment on remote data capturing, ubiquitous electronic devices with easy accessibility toward end-users are the most attractive options. In this sense, smartphones are common systems which can be easily utilized for data acquisition and their visualization [58,59,60,61]. Moreover, various software programs and their development platforms have been well established for smartphones, leading to their immediate utilization without the investment of significant resources. From this perspective, conventional user-friendly software platforms are desired, leading to our adoption of the App Inventor developed by MIT. This platform does not require a high level of programming knowledge due to its use of Scratch, a block-based programming tool [62,63]. Thus, we implemented user-interface platforms using the App Inventor. However, App Inventor does not have an easy mechanism for collecting and processing event data streams; furthermore, there are limitations in their visual presentation. On the other hand, it provides enough resources for dealing with the movement data of animals, drawing a simple two-dimensional (2D) graph, and storing the data.

### 2.3. System Design

**Figure 4:**
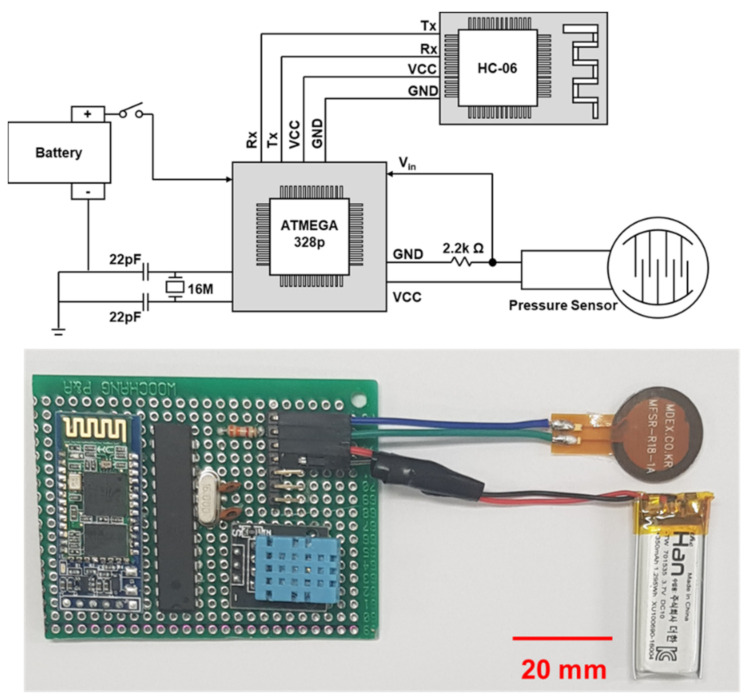
Scheme (**top**) and image (**bottom**) of the electronic platform.

Figure 4 shows the schematics for the implemented system and optical images of the platform used in this study. Pressure sensors generated output signals as a change in resistance, corresponding to the input movement. In order to read the change in resistance, the current or voltage value was returned as an input signal to the MCU. However, when the current level was generated as the output signal, an additional read module for its detection was required [64].

Thus, an ultra-low current level as the output, due to the high resistance of the sensor, could possibly lead to system errors during the reading period of output signals. On the other hand, measurement of the output voltage could be accurately detected using analog pins built into ATmega328p when the resistors were connected in series with the sensors. Therefore, the resistor node was connected to the input node of the MCU in order to detect the change in sensor output. The output voltage value of the pressure sensor node can be expressed as a ratio of load resistance (*R_L_*) to the summation of sensor resistance and load resistance (*R_S_* + *R_L_*) according to the following Equation (2):

(2) $$V_{out}=V_{in}\cdot \frac{R_{L}}{R_{L}+R_{S}},$$

where *V_out_* is the output voltage at the sensing node, *V_in_* is the applied voltage to the pressure sensor, and *R_L_* and *R_S_* are the load resistance and sensor resistance, respectively. According to Equation (2), to achieve high sensitivity at the sensing node, *R_L_* should, on average, be in the range of *R_S_*. The analog signal output from the sensor node entering the analog pin of ATega328P is converted into an 8 bit digital signal and transmitted to the smartphone application through the HC-06 antenna of the Bluetooth module.

## 3. Results

### 3.1. Method

**Figure 5:**
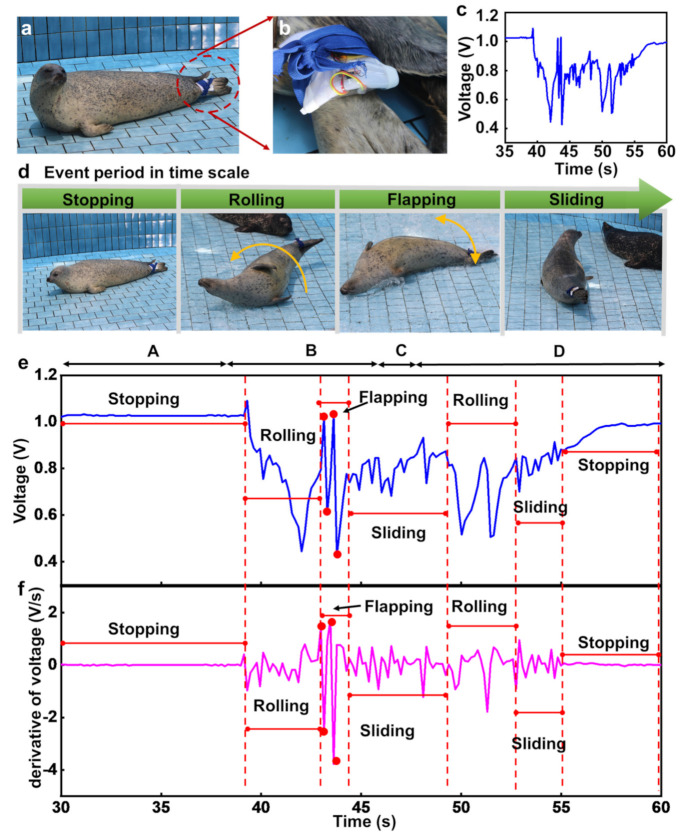
Field test process for motion capture of captive harbor seal (HS) and data analysis. (**a**) Optical image of the captive HS with biologging on the flippers. (**b**) Magnified image of the biologging system. (**c**) Voltage output plots for pressure sensors as a function of time during remote recognition of behaviors for the captive HS. (**d**) Images of the captive HS during representative motions: stopping, rolling, flapping, and sliding. (**e**) Voltage output and (**f**) its derivative as a function of time (i.e., dV/dt), corresponding to the event period.

Figure 5a shows the captive HS targeted for tracking of motion. The HS’s tail plays a role in various behaviors such as stopping, rolling, flapping, and sliding. In consideration of its physical characteristics, the HS’s flipper was deemed appropriate for the attachment of sensor platforms (Figure 5b). In addition, the device was fully covered using a PDMS encapsulant as a waterproof coating. Thereafter, for their quick and immediate attachment (or detachment) during remote recognition of HS behaviors, all sensor platforms were fixed with a rubber string (blue color), as shown in Figure 5b. Figure 5c shows the voltage signals detected for the motion of a captive HS corresponding to a time scale where the HS consecutively undertook actions freely, as shown in Figure 5d.

### 3.2. Observed Behavior

The output voltage was remotely captured using a pressure sensor corresponding to consecutive movements of the HS. Figure 5d shows the captured snapshot images, corresponding each motion of the HS, i.e., stopping (A), rolling (B), flapping (C), and sliding (D). As the HS moved freely in the pool, as shown in Figure 5d, different levels of strains around the flippers corresponding to each movement were observed, followed by simultaneous detection of output voltages from the pressure sensors. Furthermore, Figure 5e,f shows the output voltage signals and their derivatives as a function of time (i.e., dV/dt), respectively, corresponding to stopping (A), rolling (B), flapping (C), and sliding (D), followed by remaining stationary. The videos recorded for the motion of HS are provided in the Video S1 (Supplementary Materials).

**Figure 6:**
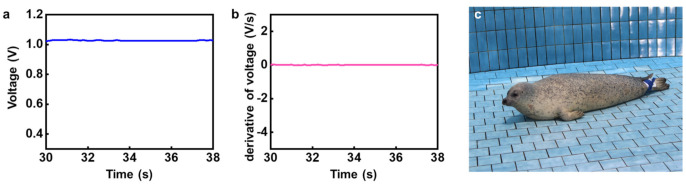
Electrical signals and optical images describing the behavior of a captive harbor seal (HS) in a stationary state: (**a**) output voltage of the pressure sensor and (**b**) its slope as a function of time; (**c**) representative image of the captive HS’s posture in a stationary state.

For a better understanding of each motion of the HS in a quantitative sense, a comparison study between the captured image and its electrical signal was performed using a frame-by-frame analysis. From this perspective, analysis of the HS’s stationary motion in the initial state is shown in Figure 6. The pressure value measured at the flipper (Figure 6a), the slope of the measured data (Figure 6b), and a snapshot (Figure 6c) are presented. As shown in Figure 6c and Video S1 (Supplementary Materials), when the seal took a rest, the output signal from the flipper showed a constant value. In the initial state, variation in the voltage output signal was constant, leading to the conclusion that the pressure sensor-enabled signal detection could reliably and precisely detect the motion of the HS.

**Figure 7:**
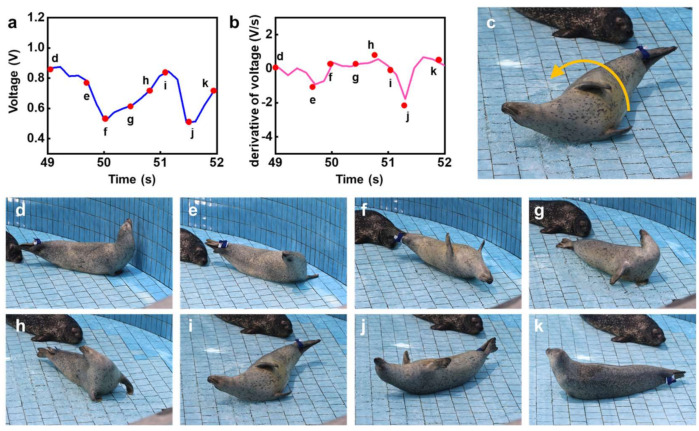
Electrical signals and images describing the consecutive behaviors of a captive harbor seal (HS) during rolling: (**a**) output voltage of the pressure sensor and (**b**) its derivative as a function of time (i.e., dV/dt); (**c**) representative image during rolling. Consecutive snapshots of the HS rolling, corresponding to each event period: (**d**) stationary moment in the initial period, taken as the starting point; (**e**) 0.7 s, (**f**) 1 s, (**g**) 1.5 s, (**h**) 1.8 s, (**i**) 2.1 s, (**j**) 2.5 s, and (**k**) 3 s after initial period.

On the other hand, for the rolling event (B), Figure 7a,b show the voltage output and its derivative as a function of time during the rolling event, respectively. Figure 7b shows variation in the derivative of voltage with respect to time. Figure 7c shows a representative image of the captive HS during the rolling event.

The distinctive phenomenon of a transition from a positive to negative value in terms of the derivative of voltage can be understood as the ending point during each rolling cycle. For example, as shown in Figure 7b, a transition from negative to positive was observed at the periods of f, i, and k. On this basis, the number of rolls could be quantitatively estimated. The consecutively captured motion images, shown in Figure 7d–k, allow reasonably validating the simple relationship between the ending points and the transition of the derivative of voltage from positive to negative. Therefore, this demonstration suggests that the pressure sensor-based electrical signal, along with quantitative and qualitative analysis, can be utilized for an understanding of motion of the captive HS, which was remotely captured via Bluetooth communication. Figure 7c–k show the variation in output signal with HS motion, with Figure 7d–h displaying the HS starting to turn (or entering into a stationary state). The corresponding derivative of voltage with respect to time would not change dramatically due to acceleration being in the same direction. However, the HS required high energy when changing its body direction, leading to high strain around its flippers, corresponding to Figure 7f,i,k.

**Figure 8:**
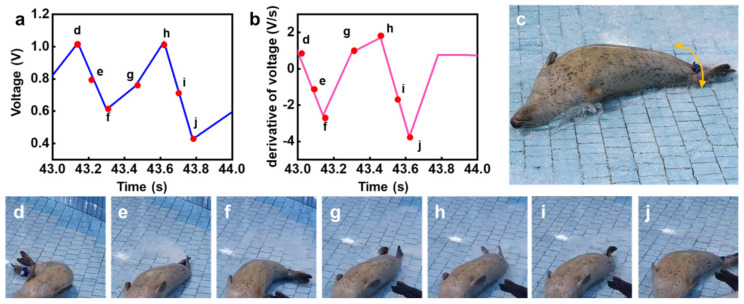
Electrical signals and images describing the flapping behavior of the HS: (**a**) output voltage of the pressure sensor and (**b**) its slope; (**c**) representative image. Images displaying the flapping motion of the seal after (**d**) 0.1 s, (**e**) 0.2 s, (**f**) 0.3 s, (**g**) 0.5 s, (**h**) 0.6 s, (**i**) 0.7 s, and (**j**) 0.8 s.

Upon completing rolling, when the HS was on its back, it started a flapping motion, as shown in Figure 8. Figure 8a,b show the output voltage and its derivative as a function of time, respectively. Figure 8c shows a representative image of the HS’s flapping motion (C). By comparing the electrical data with snapshots, as carried out above, the physical information extracted from the derivative of voltage with respect to time was obtained.

**Figure 9:**
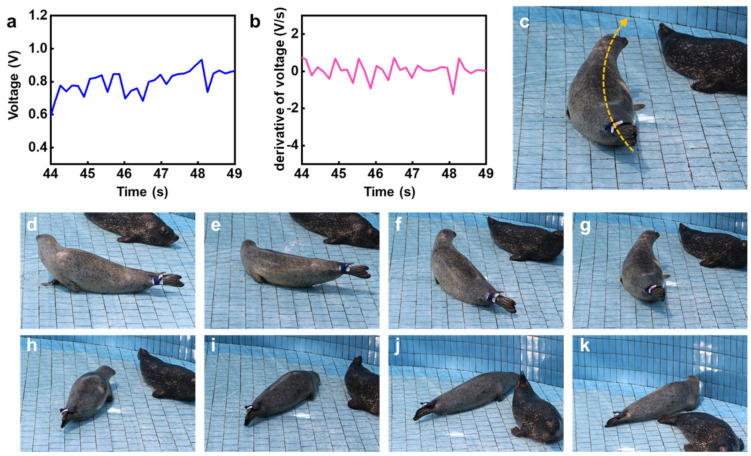
Electrical signals and images describing the sliding behavior of the HS, followed by a stopping motion. (**a**) Output voltage of the pressure sensor and (**b**) its derivative as a function of time; (**c**) representative image. Consecutive images displaying the sliding motion followed by a stationary posture after (**d**) 0.7 s, (**e**) 1.5 s, (**f**) 2.2 s, (**g**) 3.0 s, (**h**) 3.7 s, (**i**) 4.5 s, (**j**) 5.2 s, and (**k**) 6.0 s.

Due to the severe flapping motion, the internal strain around the HS’s flipper resulted in a large amplitude of 0.4–0.6 V, leading to quite a large derivative compared with the rolling motion. This distinctive and clear pattern indicates that the quantitative information of HS flapping behavior could be extracted. Moreover, the changes in polarity of the derivative of voltage, as shown in Figure 8b, allowed determining the number of flaps with respect to the location and direction of strain around the flipper. The output voltage plots for the rolling motion (B) in Figure 7a and the flapping motion (C) in Figure 8a presented similar peak values with differences in strength and amplitude. With respect to sliding (D), Figure 9a,b show the output voltage and its derivative as a function of time, respectively. As the HS slid, the voltage plots shown in Figure 9a indicate that a relatively low-amplitude voltage was observed. Thus, this behavior pattern could not be easily identified. Furthermore, the snapshots (Figure 9c–k) indicate that a stable and stationary motion was observed during the sliding motion (D), resulting in the signal from the flipper coming across as noise; however, the signal was different from that observed in the stationary state.

**Figure 10:**
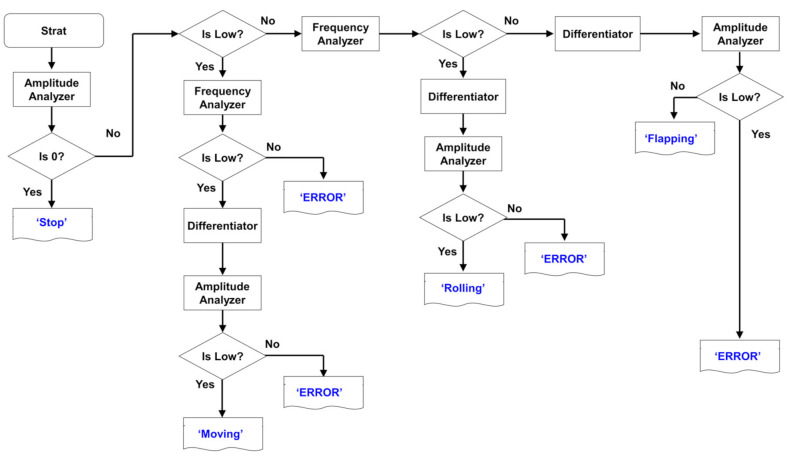
Proposed analysis algorithm with 4 functions: ’Frequency Analyzer’, ’Amplitude analyzer’, ‘Differentiator’, ‘Is Low?’. The ‘is Low?’ function has two input as analyzer output and analyzer type. During the execution of evlaution for the algorithm, the ‘Is Low?’, next to ’Frequency analyzer’, identifies if the input is greater than 1.

Moreover, we classified the behaviors on the HS, according to distinctively detected motions such as stopping, rolling, flapping, and sliding, and others. For the meaningful and practical provision on the criteria to differentiate with each motion of the HS, we developed the algorithm as shown in Figure 10. In the respective core algorithm, four kinds of functions are developed to evaluate all procedures to classify the motion. There are core functions such as amplitude analyzer, frequency analyzer, differentiator, and “is Low?”. Within experimentally obtained electrical signals, we added the Table 2 which encompass the criteria for determination of each motion in the flow chart on the basis for the development algorithm.

Based on key parameters such as frequency, amplitude, and amplitude for the signal of ‘derivative of amplitude (dV/dt)’, we extracted all reference values to differentiate each motion. Table 2 shows the most critical values to determine each motion. For one example, when the amplitude analyzer detects low value (≈0), the motion of the HS is turned out as ‘Stop’. If the amplitude analyzer is not low value, ‘Is the Low?’ function evaluate all the amplitude analyzer data. If the ’Is the Low?’ is ‘Yes’, the frequency analyzer evaluate once again. The value is turned out as ‘Yes’ via function of ‘Is the Low’, all extracted values were validated via the algorithm, leading to discernible detection in the classification on each motion for the HS. The procedure for the development of algorithm to differentiate the motion of the HS was validated in this work, which is potentially beneficial for the provision on the future extendibility toward bio-logging system with highly sensitive and accurate algorithm. Right after, evaluation on ‘Differentiator and Amplitude Analyzer’, if the value for the function of ‘Is Low?’ turns out as ‘yes’, the motion will be determined as ‘Moving’. Otherwise, the result will be turned out as ‘Error’. Likewise, ‘Rolling’ and ‘Flapping’ will be assessed as differentiated motion from the electrical signals on amplitude, frequency, and derivative of amplitude, and others. In addition, when ‘ERROR’ signal is turned out, identification and sensitivity factors are liberally adjusted in the comparison algorithm.

## 4. Conclusions

Using the well-established Arduino open-source platform along with pressure sensors, biologging systems with low cost, light weight, and remote data detectability were successfully generated, creating future research opportunities. In this study, the designed system was applied to a captive HS to gain meaningful insight into its behavior, with future potential for biologging-enabled ethology applications. In particular, the captive HS, fitted with a biologging system, was successfully used to remotely detect signals via Bluetooth communication within 7 m. The detected voltage signal from the pressure sensors and their physical representation in terms of HS behavior were validated using consecutively captured images displaying the motion of the captive HS. Right after one-time fully charging up the battery of the biologging system, the proposed platform allows effectively and remotely analyzing HS motion even in the dark with at least total operational time of 7.74 ± 2.4 h, which enables to guarantee proper operation of the biologging system. Moreover, the output voltage signals and their derivatives as a function of time provide information on the behaviors of the captive HS, such as stopping, rolling, flapping, and sliding motions. Detailed quantitative information related to consumed energy and habitual motion can be subsequently interpreted with a high level of accuracy through real-time data recorded during the motion. The presented low-cost, easily detachable biologging systems can potentially be utilized for future research opportunities such as the culturing and early detection of disease in captive sea animals. Furthermore, PDMS encapsulants can be effectively utilized for the reliable detection of signals when exposed to seawater.

As one of practical applications, remotely accessible data capturing for the wild (or captive) sea animals, monitoring on physiological signal detection during physical surgery, which might be necessary for the monitoring respiratory rate (RR), Bluetooth based monitoring will be one of powerful application to address diving response during the surgery operation. Currently, most of physiological signals are monitored via wired electronic monitoring system as the Patient Monitoring System, GE Health Care Inc. Thus, with the help of the current platforms with Bluetooth application, complicated wired connection will be relieved via reliable Bluetooth communication protocols. In addition, if the experimental environment was in the wild rather than inside the zoo, a LoRa or Cat. M1 device will be adopted for a better data transmission environment. In parallel, the pressure sensors and their hybrid sensors including lab-made epidermal sensors can conceptually enhance the capability of bio-logging systems with code-division multiple access (CDMA) based communication for addressing within several hundreds of km range. This kind of system with Cat. M1 will be tested in wild animals attached with the bio-logging systems with recently developed skills in the group. Thus, the newly developed platform can enhance the limitation on detectable range, as compared with that of Bluetooth communication platform. More importantly, PBAS (Python Based Algorithm Software) was developed in this study and basic motion behaviors were evaluated, leading to automatic detection from remotely extracted data from bio-logging system. Moreover, additional data samples are essential to improve the reliability of the developed algorithm. Therefore, the adoption of a platform that enables to accumulate a lot of data to wild animals is planned, followed by addressing this issue in an independent study. In the future study, this pressure-based approach should be compared with the commonly used inertial measurement approaches with accelerometers, gyroscopes, and magnetometers. Thus, this platform is expected to be beneficial in the field of ethology for an understanding of animal behaviors and their prediction during mating and spawning seasons.

## Figures and Tables

**Table 1:** Summary of current level and power consumption, corresponding to the microcontroller unit (MCU), Bluetooth module, temperature sensor, and pressure sensor.

	Current (mA)	Power (mW)
MCU	~7	23.1
Bluetooth	~18	59.4
Temp sensor	~0	~0
Pressure sensor	~0	~0

**Table 2:** Summary of criteria on identification for the function of ‘Is Low?’ in terms of frequency, amplitude, and amplitude for the signal of ‘derivative of amplitude (dV/dt)’.

Behavior	Frequency (Hz)	Amplitude (V)	Amplitude for the Signal of ‘Derivative of Amplitude’ (dV/dt)
Stop	-	<0.1	-
Rolling	>1	>0.5	<3
Flapping	<1	>0.5	>3
Sliding	<1	<0.5	<3

## Supplementary Materials

The following are available online at https://www.mdpi.com/2072-666X/12/3/267/s1: Video S1.: HS moving.mp4.
